# Relations between right ventricular morphology and clinical, electrical and genetic parameters in Brugada Syndrome

**DOI:** 10.1371/journal.pone.0195594

**Published:** 2018-04-13

**Authors:** Belinda Gray, Ganesh Kumar Gnanappa, Richard D. Bagnall, Giuseppe Femia, Laura Yeates, Jodie Ingles, Charlotte Burns, Rajesh Puranik, Stuart M. Grieve, Christopher Semsarian, Raymond W. Sy

**Affiliations:** 1 Royal Prince Alfred Hospital, Sydney, Australia; 2 Sydney Medical School, University of Sydney, Sydney, Australia; 3 Agnes Ginges Centre for Molecular Cardiology, Centenary Institute, Sydney, Australia; 4 Cardiovascular Magnetic Resonance, Newtown, Australia; Indiana University, UNITED STATES

## Abstract

**Background:**

Increasing evidence suggests the presence of structural changes affecting the right ventricular outflow tract (RVOT) in patients with Brugada Syndrome (BrS). The aim of this study was to characterise the RV morphology in BrS and explore associations between morphologic, clinical, electrical, and genetic parameters using non-invasive multimodality testing.

**Methods:**

Consecutive BrS patients (recruited 2013–2015) underwent clinical assessment, dedicated RV imaging using cardiac magnetic resonance (CMR) imaging (unless contra-indicated), electrical assessment (electrocardiogram, Holter monitoring, signal-averaged ECG[SAECG]) and genotyping. Morphologic data were compared to matched control and unmatched ARVC (arrhythmogenic right ventricular cardiomyopathy) cohorts, and potential associations between morphologic parameters and other variables were explored.

**Results:**

BrS patients (n = 42, male 86%, age 46±12 years) exhibited normal global RV volume and function, comparable to control, in contrast to significantly larger, impaired RVs in ARVC cohort (RVESV p = 0.0001; RVEDV p<0.0001, RVEF p = 0.002). Compared with control, BrS patients exhibited larger RVOT volumes (7.4 ± 0.7 vs 5.8 ± 0.7 mL/m^2^, p<0.0001) and wall motion abnormalities (RWMA) (31% vs 0%, p = 0.005); compared with ARVC cohort, the RVOT volumes were similar (7.4 ± 0.7 vs, 8.1 ± 1.7, p = 0.52) and there were less RWMA (31% vs 76%, p = 0.01). Overall 67% BrS patients had abnormal RVOT morphology. Patients with abnormal RVOT tended to be older (48 ± 12 y vs 41 ± 12y, p = 0.06). Rare genetic variants were only observed in patients with abnormal RVOT morphology (36% vs 0%, p = 0.02).

**Conclusions:**

Patients with BrS frequently exhibit structural abnormalities localised to the RVOT and these changes may be age- and gene-dependent.

## Introduction

Brugada Syndrome (BrS) is an inherited arrhythmia syndrome characterised by coved-shaped ST elevation in the right precordial leads on 12-lead electrocardiogram (ECG)[1–3]. BrS has typically been considered a primary inherited channelopathy, most commonly due to loss of function of the inward sodium current, in the absence of overt structural heart disease. However, there is increasing evidence that BrS may represent a heterogeneous group of disorders with a unifying ECG abnormality[4]. Recent radiological and histological studies have highlighted the BrS arrhythmic substrate originates from the right ventricular outflow tract (RVOT) [5–9]. Several groups have also proposed that the arrhythmic substrate and ECG changes in BrS may be ameliorated by RVOT ablation[10–12]. There are few studies that have systematically explored the association between structural abnormalities and the electrical and genetic profile of patients with BrS[13, 14].

Evaluation of patients with BrS has typically focussed on clinical, genetic and electrical parameters based on ECG and/or EP testing[2, 3, 15]. However, given the mounting evidence that BrS may not be a pure electrical disease, the inclusion of adjunctive investigations such as dedicated RV imaging, signal-averaged ECG (SAECG), and 12-lead Holter monitoring as part of a multimodality assessment approach may improve our understanding of BrS. The aim of this study was to use multimodality assessment to characterise the structural pathophysiology in BrS and specifically explore potential associations between morphologic changes and clinical, electrical, and genetic parameters.

## Methods

### Patient selection and baseline assessment

From July 2013 to December 2015, consecutive BrS patients were recruited from Genetic Heart Disease Clinics at Royal Prince Alfred Hospital and Concord Repatriation General Hospital in Sydney, Australia, as well as the Australian Genetic Heart Disease Registry[16]. All patients had a definite diagnosis BrS according to published criteria[2]. The study was approved by the Sydney Local Health District Ethics Review Committee, Australia.

Patients underwent clinical review and ECG with precordial leads placed in standard position and between 2^nd^ and 4^th^ ICS. In addition to measuring standard baseline ECG intervals (e.g. PR, QRS, QTc) we also specifically interrogated ECGs for the presence of fragmented-QRS [defined as ≥4 spikes in one lead or ≥8 spikes in all leads V1-V3][17] and inferolateral ST change [defined as prominent J-point elevation of at least 1mm in any inferolateral lead][18]. Abnormal findings required concordance between 2 independent cardiologists (BG/RS). After enrolment, all patients were prospectively followed and reviewed annually or following a clinical event. All patients with suspected arrhythmic syncope underwent careful history by two independent physicians (BG, RS).

### Dedicated RVOT imaging and quantitative assessment using Cine MRI

All eligible patients were referred for CMR imaging with dedicated RV and RVOT analysis. Patients with contraindications to CMR (ICDs *in situ* or severe claustrophobia) were referred for echocardiogram with quantitative analysis of the RV and RVOT. CMR was performed using 1.5T scanner (GE Medical Systems). Detailed CMR protocol is found in **S1 Text, S1 Fig and S2 Fig**. Echocardiogram was performed with RVOT measurements performed in parasternal short axis view, in accordance with the 2010 Task Force Criteria for arrhythmogenic right ventricular cardiomyopathy (ARVC) (**S2 Fig**) [19–21]. Abnormal RVOT was defined as the presence of RVOT regional wall motion abnormalities (RWMA; defined as akinesis, dyskinesis) or an RVOT diameter >25mm[22]. RV morphological parameters from BrS cohort were compared to those in an age- (within 5 years) and gender-matched healthy control cohort without heart disease, as well as an unmatched cohort of consecutive patients with ARVC who were referred to our clinic during the study period. All patients in the ARVC cohort fulfilled published criteria for definite diagnosis[20] and the two most common presentations were documented ventricular arrhythmia (47%) and family history of premature sudden death or ARVC (23%).

### 12-lead Holter monitoring & signal averaged ECG

Patients underwent 12-lead 24-hour Holter assessment with chest leads placed between the parasternal 2^nd^ and 4^th^ ICS. The methods for collection and analysis of Holter data have been previously described[23]. Only coved-type ST-elevation ≥2mm was used for ST analysis. “Spatial burden” was calculated by number of precordial leads demonstrating diagnostic ST elevation at any time-point. “Global burden” was defined as the summed ST elevation across all precordial leads during 24-hours (in mm). “Temporal burden” was defined as the total time duration (in minutes) with type 1 pattern over the 24-hour period.

The presence of late potentials was defined as ≥1 out of the following 3 criteria: filtered QRS duration >114ms, terminal (last 40ms) QRS root mean square <20μV or low amplitude signal (under 40 μV) duration >38ms[20].

### Genetic testing

The majority of patients (88%) were referred for research-based genetic testing including previously reported cardiac arrhythmia, ARVC and other cardiomyopathy genes. This involved cardiac gene panel testing in 21 patients [Illumina Trusight extended arrhythmia/cardiomyopathy 174 gene panel (10 patients) or Blueprint Genetics (Finland) 133 heart gene panel (11 patients)] or whole exome sequencing (Macrogen, Korea) in 16 patients. A minority of patients (12%) were referred to our clinic with known genetic variants in BrS1-23 identified on prior commercial testing. Any rare variants (allele frequency <0.02% in the Exome Aggregation Consortium database, http://exac.broadinstitue.org) were then assessed for pathogenicity using modified ACMG criteria[24] (see ClinVar, Agnes Ginges Centre for Molecular Cardiology variant assessment and assertion criteria; https://submit.ncbi.nlm.nih.gov/ft/byid/djgybgii/mdi-5363_505375_agnesginges_variantassess_clinvar.pdf).

### Statistical analysis

Statistical analyses were carried out using SPSS (Version 23) and GraphPad Prism 7. Continuous variables were assessed using unpaired T-tests and one-way analysis of variances for > 2 groups. If variables were not normally distributed they were summarized with medians and interquartile ranges and compared with Mann-Whitney Test. Categorical variables were compared using chi-square and Fisher’s exact tests. Linear regression analysis was performed to study the relation between morphologic parameters, and continuous electrical parameters. Significance was set at a two-sided p-value of <0.05.

## Results

### Baseline characteristic

A total of 42 patients were recruited. Baseline characteristics are shown in **Table 1**. The majority of the patients were male (n = 36, 86%), mean age at diagnosis was 46 ± 12 years and mean follow-up was 2.2 ± 2.0 years. Most patients were probands (n = 37, 88%), 10 (24%) had a family history of SCD or ACA and, 20 (48%) were classified as having a “spontaneous BrS pattern” on the baseline ECG or Holter monitoring. There were 11 patients (26%) who were symptomatic at recruitment [n = 5 (12%) ACA, n = 6 (14%) syncope], all of whom had ICDs *in situ*.

**Table 1:** Baseline characteristics.

Characteristic	Value*
Male	36 (86)
Age at diagnosis (yrs)	46 ± 12
Follow up (yrs)	2.2 ± 2.0
Spontaneous type 1 pattern on ECG or Holter^20^	20 (48)
Proband	37 (88)
History of ACA	5 (12)
History of Syncope	6 (14)
Shanghai Score^3^	3.7 ± 1.7
Family History of sudden cardiac death/ACA < 45 years	10 (24)
ICD at recruitment	11 (26)
**Previous Electrophysiology study**	4 (10)
Ventricular Effective Refractory Period <200ms	0 (0)
Inducible VF/VT	1(25)
**Genetic Testing Result**	
Pathogenic/Likely Pathogenic	7(17)
Any rare variant	10 (24)

ACA- aborted cardiac arrest.

*values are mean ± SD or n (%).

Baseline morphologic characteristics are shown in **Table 2**. CMR imaging was performed in 29 (69%) patients. Echocardiogram with dedicated RV imaging was performed in remaining 13 (31%) patients due to contraindications to CMR (11 ICD, 2 claustrophobia). Overall biventricular size and function were normal. One patient (3%) had an indexed RVEDV beyond the normal range for the laboratory, and no patients had RVEF ≤40%. Mean RVOT diameter was 25 ± 4mm.

**Table 2:** Morphologic and electrical characteristics.

Characteristic	Value*
***Imaging***	
LVESVI (mL/m2)^+^	26 ± 8
LVEDVI (mL/m2)^+^	70 ± 19
RVESVI (mL/m2)^+^	35 ± 12
RVEDVI (mL/m2)^+^	80 ± 26
LVEF (%)*	64 ± 7*
RVEF (%)*	56 ± 6*
RVOT dimension (overall)*	25 ± 4*
-*CMR dimension*	26 ± 4
-*TTE dimension*	25 ± 3
RVOT length	33 ± 5
RVOT volume	14 ± 2
RVOTI: RVEDVI ratio	0.11 ± 0.04
RVOT wall motion abnormality*	12 (29)*
***ECG***	
Heart rate (bpm)	68 ± 12
PR (ms)	168 ± 25
QRS(ms)	115 ± 17
QTc (ms)	400 ± 20
Inferolateral ST change	4 (10)
Fragmented QRS	5 (12)
***12 lead 24 hour Holter Monitoring***	
Spatial burden: Maximum number of leads[23]	1.1 ± 1.6
Global burden: Total ST elevation (mm)[23]	102 ± 266
Temporal burden: Total Time Burden ST elevation (min)[23]	512 ± 1330
SDNN (ms)	135 ± 40
rMSSD (ms)	38 ± 18
SDANN (ms)	97 ± 46
pNN50 (%)	11 ± 10
Premature ventricular contractions	149 ± 452
***SAECG***	
Late Potentials	15 (36)
Positive Late Potential Count	0.7 ± 1
HF QRS duration	102 ± 14
RMS last 40ms	32 ± 21
Duration <40μV	33 ± 10

LV/RVES/DVI- left/right ventricular end-systolic/diastolic volume indexed, LV/RVEF- left/right ventricular ejection fraction, QTc- corrected QT interval, SD(A)NN- SD of normal (averaged) RR intervals, rMMSD- root mean square of successive normal sinus RR interval difference, pnn50- % normal sinus RR intervals >50ms.

(Values are mean ± or SD or n(%)).

^+^Indexed.

*Includes echo if no CMR.

On baseline ECG, four patients (10%) had inferolateral ST change and five patients (12%) fQRS (**Table 2**). Holter Monitoring demonstrated mean summed type 1 ST elevation of 102 ± 266mm over 24-hour period. The mean time with type 1 pattern ST elevation (i.e. temporal burden) was 512 ± 1330 minutes, and mean spatial burden was 1.1 ± 1.6 leads. The overall PVC burden was low (149 ± 452 beats) and no patients demonstrated VT. Late potentials were observed in 15 patients (36%).

Rare variants in BrS genes were identified in 24% of patients, and the variant was defined as pathogenic or likely pathogenic in 17% **(Table 1).** Specific genetic variants are described in **S1 Table**.

### Morphologic RVOT abnormalities in Brugada syndrome

The imaging data from the BrS cohort were compared to the ARVC and control groups (**Table 3**). BrS patients had similar global left ventricular volume and function as both ARVC and control groups, except for lower LVEDV compared to ARVC cohort (p = 0.04).

**Table 3:** Comparison of CMR data.

Characteristic	Brugada cohort n = 29	ARVC cohort n = 17	Control Group n = 29	p value
Age at scan	48 ± 12	39 ± 17	48 ± 10	0.10
Male sex	25 (86)	11 (65)	17 (81)	0.22
Body surface area	1.9 ± 0.2	1.9 ± 0.3	2.1 ± 0.2	0.08
LVESVI (mL/m^2^)^+^	25 ± 8	31 ± 11	27 ± 9	0.11
LVEDVI (mL/m^2^) ^+^	67 ± 14	79 ± 18	73 ± 13	0.04*
RVESVI (mL/m^2^) ^+^	33 ± 9	60 ± 38	37 ± 7	0.0001*
RVEDVI (mL/m^2^) ^+^	75 ± 16	111 ± 44	83 ± 12	<0.0001*
LVEF (%)	64 ± 7	61 ± 8	64 ± 6	0.36
RVEF (%)	56 ± 6	49 ± 10	56 ± 5	0.002*
RVOTDI^+^ (mm/m^2^)	13.4 ± 2.1	14.6 ± 1.5	13.0 ± 1.4	0.03*
RVOTVI^+^ (mL/m^2^)	7.4 ± 0.7	8.1 ± 1.7	5.7 ± 0.6	<0.0001*
RVOT WMA	9 (31)	13 (76)	0 (0)	<0.0001*
RVOTVI:RVEDVI	0.11 ± 0.04	0.07 ± 0.02	0.07 ± 0.01	<0.0001*
RV late gadolinium enhancement	0	0	0	0.93

[Results are n (%) or mean ± SD (one-way ANOVA)].

^+^Indexed.

*p<0.05.

LV/RVES/DVI- left/right ventricular end-systolic/diastolic volume indexed, LV/RVEF- left/right ventricular ejection fraction, RVOTD/VI- right ventricular outflow tract diameter/volume indexed.

**Fig 1:**
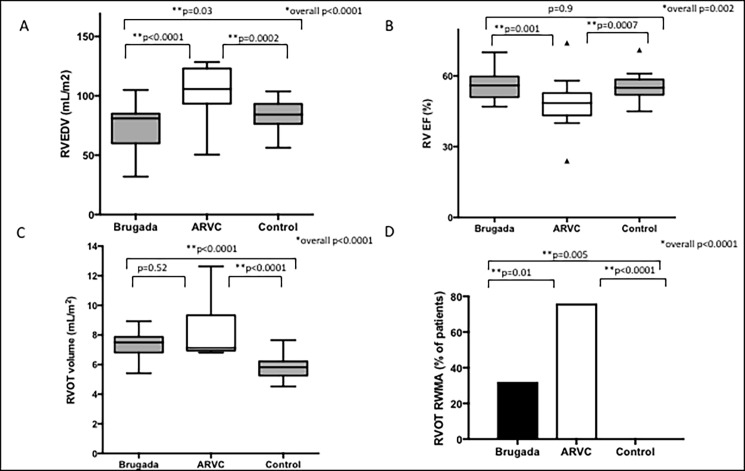
Morphology comparison between BrS, ARVC and control cohort. **Box and whiskers plot of (A) RVEDV (B) RVEF (C) RVOT volume; column graph of (D) RVOT RWMA in BrS compared with control and ARVC cohorts.** Box and whiskers values are median (IQR). Whiskers include values within 1.5 IQR of the nearest quartile. Boxes are based on Tukey’s Hinges. *overall comparisons RVEDV/RVEF/RVOT volume one-way ANOVA; RVOT RWMA Fishers-exact test. **column comparisons Mann-Whitney test.

BrS global RV volume and function was comparable to control except for lower RVEDV (p = 0.03), though probably not clinically relevant because both groups were within normal ranges. ARVC patients had larger global RV volumes and reduced systolic function when compared to both BrS patients and controls (RVESV = 33 ± 9 [BrS] vs 37 ± 7 [control] vs 60 ± 38 [ARVC] mL/m^2^, p = 0.0001; RVEDV = 75 ± 16 [BrS] vs 83 ± 12 [control] vs 111 ± 44 [ARVC] mL/m^2^, p<0.0001, one-way ANOVA **Fig 1A**; RVEF = 56 ± 6% [BrS] vs 56 ± 5% [control] vs 49 ± 10% [ARVC], overall p = 0.002 one-way ANOVA [BrS vs control p = 0.9, BrS vs ARVC p = 0.001, Mann-Whitney test]**, Fig 1B**).

When specifically assessing function and size of the RVOT, BrS patients were found to have significantly greater indexed RVOT volumes compared with control (7.4 ± 0.7 vs 5.7 ± 0.6 mL/m^2^, p<0.0001) and similar to the ARVC cohort (7.4 ± 0.7 vs, 8.1 ± 1.7, p = 0.52 Mann-Whitney test, **Fig 1C**). BrS patients exhibited significantly more focal RVOT regional wall motion abnormalities than the control group (31% vs 0%, p = 0.005) but less than the ARVC cohort (31% vs 76%, p = 0.01, overall p<0.0001, Fishers Exact Test **Fig 1D**). BrS patients had significantly greater indexed RVOT volume:RVEDV ratios than ARVC and control (RVOTVI:RVEDVI = 0.11 ± 0.04 [BrS] vs 0.07 ± 0.02 [ARVC] vs 0.07 ± 0.01 [control], p<0.0001, overall one-way ANOVA; BrS vs control p<0.0001 Mann-Whitney test, BrS vs ARVC p<0.0001 Mann-Whitney test). The RVOTVI:RVEDVI ratio in the ARVC cohort was not significantly different to the control group (ARVC vs control p = 0.92, Mann-Whitney test). There was no evidence of right or left ventricular late gadolinium enhancement (LGE) in any group. None of the BrS patients fulfilled major or minor imaging criteria for ARVC [21].

### Relation between morphologic parameters, clinical characteristics and genetic profile

Using dedicated RVOT imaging, 28 BrS patients (67%) exhibited abnormal RVOT morphology; 12 patients (29%) with regional RVOT wall motion abnormality, 19 patients (45%) with RVOT diameter >25mm, (3 patients with both). The baseline characteristics and investigations of BrS patients according to RVOT morphology are shown in **Table 4**. There were no significant differences in the baseline clinical characteristics between the two groups, although patients with abnormal RVOT tended to be older (48 ± 12 y vs 41 ± 12y, p = 0.06). There was no association between abnormal morphology and clinical events (OR [95% CI] = 1.2 [0.31–4.5], p = 0.82).

**Table 4:** Association between morphological abnormality and clinical and genetic characteristics.

Characteristic	RVOT normal n = 14	RVOT abnormal n = 28	p value
Male sex	13 (93)	23 (82)	0.65
Age at diagnosis	41 ± 12	48 ± 12	0.06
Asian ethnicity	5 (36)	9 (32)	1.0
Family history of SCD/ACA	3 (21)	7 (25)	1.0
Proband	13 (93)	24 (86)	0.65
Shanghai Score	4 ± 1.6	3.7 ± 1.7	0.68
Clinical events:			
Hx of ACA	1 (7)	4 (14)	0.65
Hx of syncope	3 (21)	3 (11)	0.38
Follow up (years)	2.5 ± 2.5	2.1 ± 1.6	0.59
**Genetic Testing**			
Rare variants ^+^	0 (0)	10 (36)	0.02
- *Pathogenic/ likely pathogenic*	0 (0)	7 (25)	0.08
- *VUS*	0 (0)	3 (11)	0.54

[Results are n (%) or mean ± SD].

^+^Rarity defined as MAF<0.02% ExAC.

Rare variants identified in a BrS gene were only observed in patients with abnormal RVOT morphology (36% vs 0%, p = 0.02). Pathogenic variants in sodium channel genes gene were also only observed in patients with abnormal RVOT morphology (25% vs 0%, p = 0.08).

### Relation between morphologic parameters and electrical parameters

The presence of a spontaneous type 1 ECG pattern on ECG or Holter monitoring was associated with a lower RVEF (53.1 ± 4.1% vs 57.8 ± 6.0%; p = 0.03) but similar RVOT diameter (12.8 ± 2.1 vs 13.7 ± 2.0 mm; p = 0.25). The relation between RV morphology and f-QRS and inferolateral ST change could not be evaluated because of the low number of patients with these electrical abnormalities. Based on SAECG, patients with late potentials had a lower RVEF (53.7 ± 7.4 vs 57.5 ± 4.7; p = 0.06) but similar RVOT diameter (12.6 ± 2.2 vs 13.9 ± 2.0 mm; p = 0.17).

There was also weak statistical correlation between lower RVEF and increased QRS duration (R^2^ 0.32, p = 0.002), but no correlation between morphologic parameters and PR interval (**S2 Table**). Similarly, there was weak statistical correlation between increased RVESV and an increased spatial (R^2^ 0.26, p = 0.009) and global (R^2^ 0.26, p = 0.04) burden of Type 1 ECG changes. There was no correlation between morphologic parameters and temporal burden of Type 1 ECG changes.

## Discussion

Potential relations between morphological abnormalities and clinical, genetic and electrical characteristics in BrS were explored using multimodality non-invasive assessment including quantitative evaluation of the RV. The key findings of this study are: (1) patients with BrS had preserved overall RV volume and function but frequently exhibit localised abnormalities in the RVOT when compared with a matched control group; (2) the development of morphological abnormalities may be related to age and rare genetic variants, and; (3) patients with spontaneous type 1 ECG changes may exhibit subclinical RV dysfunction (lower RVEF) while other minor correlations between morphologic abnormalities and non-invasive electrical parameters were also observed.

### Morphologic RVOT abnormalities in Brugada syndrome

Several CMR studies have suggested RVOT morphological abnormalities in BrS patients[6, 25]. Studies have demonstrated significantly larger RVOT area and reduced RVOT ejection fraction in BrS patients compared with normal controls[5, 6, 13, 14]. However this has not been a universal finding[26]. Using quantitative analysis of the RV and RVOT, we confirmed that the overall incidence of RVOT morphologic abnormalities was high, being 67% in our cohort. The present study was unique in that it not only compared BrS cohort against age-matched controls, but also consecutive patients with definite ARVC referred to our clinic over the same time period. By doing so, we were able to demonstrate that BrS is differentiated from the normal population by the presence of increased RVOT volume and abnormal RVOT function, but that BrS is also differentiated from classic ARVC by the absence of global RV dilatation or dysfunction. The potential overlap between ARVC and BrS as part of an arrhythmogenic cardiomyopathy spectrum, was first highlighted in a series of young sudden death victims in Italy whereby 14% of the patients had a previously documented type 1 BrS ECG pattern, of whom all except one had ARVC at post-mortem[27]. Cerrone et al identified coexistence of *PKP2* mutations and sodium channel dysfunction in BrS patients with no overt ARVC phenotype. The sodium channel deficiency was due to reduced number of channels at the intercalated disk and increased microtubular separation at a cellular level[28]. Patients with ARVC have been shown to demonstrate BrS-like ECGs spontaneously and following Ajmaline provocation[29, 30]. Moreover, patients with ARVC may harbour rare SCN5A variants[31].

LGE was not observed in either our BrS or ARVC cohorts. Other groups have also described a low incidence of LGE in ARVC[32]. Basitaenen et al recently reported that 8% of their BrS patients exhibited LGE (most often in the LV)[14], reflecting the heterogeneous substrate in BrS. It is possible that the morphologic changes in BrS may be under-estimated in the present study because patients with the most severe clinical phenotype had ICDs, which prohibited CMR evaluation.

### Relation between abnormal RVOT morphology and clinical and genetic profile

Royer et al first demonstrated how ion channel defects can lead to structurally abnormal hearts, demonstrating aged-related changes in *SCN5A* knockout mouse models whereby the old but not the young *SCN5A*-mutant mice showed extensive myocardial fibrosis with heterogeneous expression of connexin-43[33]. Previous investigators have also found that *SCN5A*-mutation carriers have larger biventricular volumes and lower LVEF compared to *SCN5A*-mutation negative patients or healthy volunteers[13]. More recently, *SCN5A*-mutation carriers have also been demonstrated to have larger RV volumes and lower RVEF compared to mutation negative patients or controls[7]. In the present study, we also demonstrated that rare variants in BrS-associated genes (especially *SCN5A*) were over-represented in patients with abnormal RVOT morphology, and such variants were not found in patients with normal RVOT morphology. Coronel et al explored the substrate of an explanted RVOT following cardiac transplantation in a patient with *SCN5A* mutation. They showed RVOT histological evidence of hypertrophy and fibrosis[10]. Additionally, Frustaci et al showed right ventricular myopathic changes in association with *SCN5A* mutations[8]. Indeed *SCN5A*-mutations have been implicated in clinical phenotypes beyond BrS such as dilated cardiomyopathy and ARVC[31, 34]. These findings suggest a unifying pathogenic basis for disease whereby genetic mutations may result in cardiac channel dysfunction as well as subtle RVOT structural changes in many patients with BrS. Nevertheless it is worth noting that RVOT fibrosis has also been identified in BrS patients irrespective of mutation status again highlighting the likely underlying structural RVOT abnormalities of BrS[9]. In addition, there is increasing evidence to suggest a possible oligogenic basis to BrS with genotype-phenotype mismatch identified even in families with presumed pathogenic SCN5A mutations[35, 36].

We also observed that patients with RVOT abnormalities were on average 7 years older than patients without such abnormalities. One may speculate that structural manifestations are age-dependent and may be preceded by the electrical phenotype. Cardiac MRI is currently the gold standard for imaging the RV and RVOT because of its high spatial resolution, superior tissue characterization and ability to reproducibly quantify local RVOT volume and function. However, there remain limitations in the assessment of ultrastructural changes that may precede detectable morphologic abnormalities using existing imaging technology. For example, a recent pathological study assessing the arrhythmic substrate in post-mortem BrS hearts identified increased collagen and connexin-43 as markers of fibrosis, with the highest degree of fibrosis in the RVOT[9]. Therefore, the absence of structural RVOT changes on CMR in younger patients with BrS may not equate to the absence of an arrhythmogenic substrate in this region but rather our inability to detect early changes with current imaging tests. Serial prospective imaging studies may allow us to define the evolution of structural abnormalities in BrS.

### Relation between abnormal RVOT morphology and electrical manifestations

There have been multiple recent studies observing the importance of the RVOT in arrhythmogenesis in BrS[8–12, 37]. This has led some investigators to consider epicardial RVOT ablation as a means of ‘curing’ BrS [8, 10–12]. However, there are only a few studies that have carefully explored the association between structural abnormalities and electrical manifestations. Veltmann et al correlated the anatomical location of the RVOT on CMR imaging with the localised ECG changes[38]. Nadamanee et al showed correlation between the presence of fibrosis and abnormal late fractionated potentials indicative of slowed conduction in the RVOT region of patients with BrS[9]. Papavassiliu et al also showed that patients with spontaneous type 1 ECG pattern were more likely to have enlarged RVOT area, larger RV end-systolic volumes, lower LV and RV ejection fraction[25]. Association between PR and QRS prolongation on surface ECG with lower biventricular function have also been observed in patients with BrS[13].

The present study provided additional insights into potential relations between morphologic abnormalities and electrical manifestations of disease. We confirmed that the presence of a spontaneous type 1 ECG pattern was associated with a lower RVEF. We also found that BrS patients with late potentials tended to have a lower RVEF. Weak statistical correlation was also observed between QRS prolongation and lower RVEF, as well as between increased spatial and global burden on Holter analysis and an increased RVESV.

In totality, these findings further support recent observations of a focal arrhythmic substrate harboured in the RVOT that may be amenable to ablation in some patients with BrS. Invasive electroanatomical mapping and novel non-invasive electrocardiographic imaging methods may provide further insight into correlations between electrophysiological substrate (eg. areas of abnormal slow conduction or low voltage) and structural changes in the RVOT detected on CMR[39, 40]. Finally, future studies should evaluate whether pre-procedural CMR may assist in the identification of specific BrS patients who are more likely to benefit from epicardial mapping and ablation, notwithstanding the potential limitations of the MRI in detecting early electrical and ultrastructural changes.

### Limitations

A limitation of our study is the small size of the study, despite recruitment of consecutive patients from multiple Australian centres. The preliminary results of the present study should ideally be validated in a larger multicentre study. Due to contraindications (largely, the presence of ICDs), not all patients had CMR with some having RVOT focused echocardiograms. Recent data from Gotschy et al showed that the parasternal short axis view on echocardiogram and CMR (as used in the present study) resulted in RVOT measurements with reasonable correlation and excellent inter- and intra-reader reproducibility[19]. Another potential limitation is variability in the observation of RVOT wall motion abnormalities as highlighted by Teske et al, and therefore wall motion abnormalties in the present study required confirmation by two independent observers [41]. The lack of invasive RVOT electrophysiological data also needs to be acknowledged. At the time of study inception, the results from PRELUDE made ethics approval for routine invasive assessment on clinical grounds challenging[15]. With recent renewed interest, it is possible that the addition of invasive electrophysiologic parameters may further enhance comprehensive multimodality assessment.

### Conclusions

This study demonstrated a high incidence of RVOT morphologic abnormalities in BrS as well as important relations between such abnormalities and clinical, genetic and electrical manifestations of disease. It confirmed that BrS is indeed a heterogeneous disorder covering the spectrum of channelopathy and “cardiomyopathy” with the abnormalities anatomically localised to the RVOT in many cases.

## Supporting information

S1 Table Rare variants seen in cohort and ACMG classification. (DOCX)

S2 Table Correlation between morphologic and electrical parameters. (DOCX)

S1 Fig Example of right ventricular outflow tract volumetric measurement with cardiac magnetic resonance imaging. (TIFF)

S2 Fig Example of right ventricular outflow tract measurement using (A) cardiac magnetic resonance imaging and (B) echocardiogram.
(TIFF)

S1 Text Supplementary methods. (DOCX)

S2 Text Minimum data set CMR and echocardiogram ssssmeasurements. (PDF)

## Abbreviations

RVOT: right ventricular outflow tract
BrS: Brugada Syndrome
CMR: cardiac magnetic resonance
ECG: electrocardiogram
SAECG: signal-averaged electrocardiogram
CMR: cardiac magnetic resonance
RVESV/RVEDV: right ventricular end systolic/diastolic volume
RVEF: right ventricular ejection fraction
RWMA: regional wall motion abnormalities
