# Supplementary material for: Relations between right ventricular morphology and clinical, electrical and genetic parameters in Brugada Syndrome
Source: PLoS One. 2018 Apr 13;13(4):e0195594. doi: 10.1371/journal.pone.0195594 (PMC5898761; doi:10.1371/journal.pone.0195594)
Supplement: S1 Table — (DOCX) [file pone.0195594.s001.docx]

**S1 Table :**

| **Gene** | **Mutation** | **Classification** |
| --- | --- | --- |
| *SCN5A* | p.Phe861Trpfs*90 | Pathogenic |
| *SCN1B* | p.Trp179* | Pathogenic~ |
| *SCN5A* | p.Gly752Arg | Pathogenic |
| *SCN5A* | p.Arg1362fs | Pathogenic |
| *SCN5A* | p.Gln646ArgfsX5* | Pathogenic |
| *SCN5A* | p.Gly1743Glu | Likely Pathogenic |
| *SCN5A* | p.Lys317Glu | VUS |
| *CACNA2D1* | p.Val258Met | VUS |
| *KCNJ8* | p.Met110Leu | VUS |

* Rare variants defined as allele frequency <0.02% in the Exome Aggregation Consortium database, http://exac.broadinstitue.org

^+^ Total families =9

^~^*SCN1B* variant was seen in two related study participants

Table Legend: VUS- variant of uncertain significance
