# Supplementary material for: Relations between right ventricular morphology and clinical, electrical and genetic parameters in Brugada Syndrome
Source: PLoS One. 2018 Apr 13;13(4):e0195594. doi: 10.1371/journal.pone.0195594 (PMC5898761; doi:10.1371/journal.pone.0195594)
Supplement: S2 Table — (DOCX) [file pone.0195594.s002.docx]

**S2 Table**

|  | Correlation coefficient, R^2^ | P value |
| --- | --- | --- |
| ***Baseline ECG:*** |  |  |
| PR vs RVESV | 0.001 | 0.86 |
| PR vs RVEDV | 0.002 | 0.82 |
| PR vs RVEF | 0.004 | 0.92 |
| PR vs indexed RVOT diameter | 0.01 | 0.58 |
| PR vs RVOT volume | 0.06 | 0.26 |
|  |  |  |
| QRS vs RVESV | 0.09 | 0.14 |
| QRS vs RVEDV | 0.0001 | 0.95 |
| QRS vs RVEF | 0.32 | 0.002 |
| QRS vs indexed RVOT diameter | 0.14 | 0.06 |
| QRS vs RVOT volume | 0.002 | 0.82 |
|  |  |  |
| ***Holter monitoring*** |  |  |
|  |  |  |
| Spatial burden* vs RVESV | 0.26 | 0.009 |
| Spatial burden vs RVEDV | 0.12 | 0.09 |
| Spatial burden vs RVEF | 0.15 | 0.0497 |
| Spatial burden vs indexed RVOT diameter | 0.00009 | 0.96 |
| Spatial burden vs RVOT volume | 0.02 | 0.48 |
|  |  |  |
| Global burden* vs RVESV | 0.26 | 0.04 |
| Global burden vs RVEDV | 0.06 | 0.24 |
| Global burden vs RVEF | 0.12 | 0.08 |
| Global burden vs indexed RVOT diameter | 0.008 | 0.66 |
| Global burden vs RVOT volume | 0.002 | 0.83 |
|  |  |  |
| Temporal burden* vs RVESV | 0.05 | 0.29 |
| Temporal burden vs RVEDV | 0.02 | 0.5 |
| Temporal burden vs RVEF | 0.03 | 0.39 |
| Temporal burden vs indexed RVOT diameter | 0.001 | 0.86 |
| Temporal burden vs RVOT volume | 0.004 | 0.76 |

Analysis by linear regression

* As defined in *Methods*
