# Supplementary figures and images for: Relations between right ventricular morphology and clinical, electrical and genetic parameters in Brugada Syndrome
Source: PLoS One. 2018 Apr 13;13(4):e0195594. doi: 10.1371/journal.pone.0195594 (PMC5898761; doi:10.1371/journal.pone.0195594)

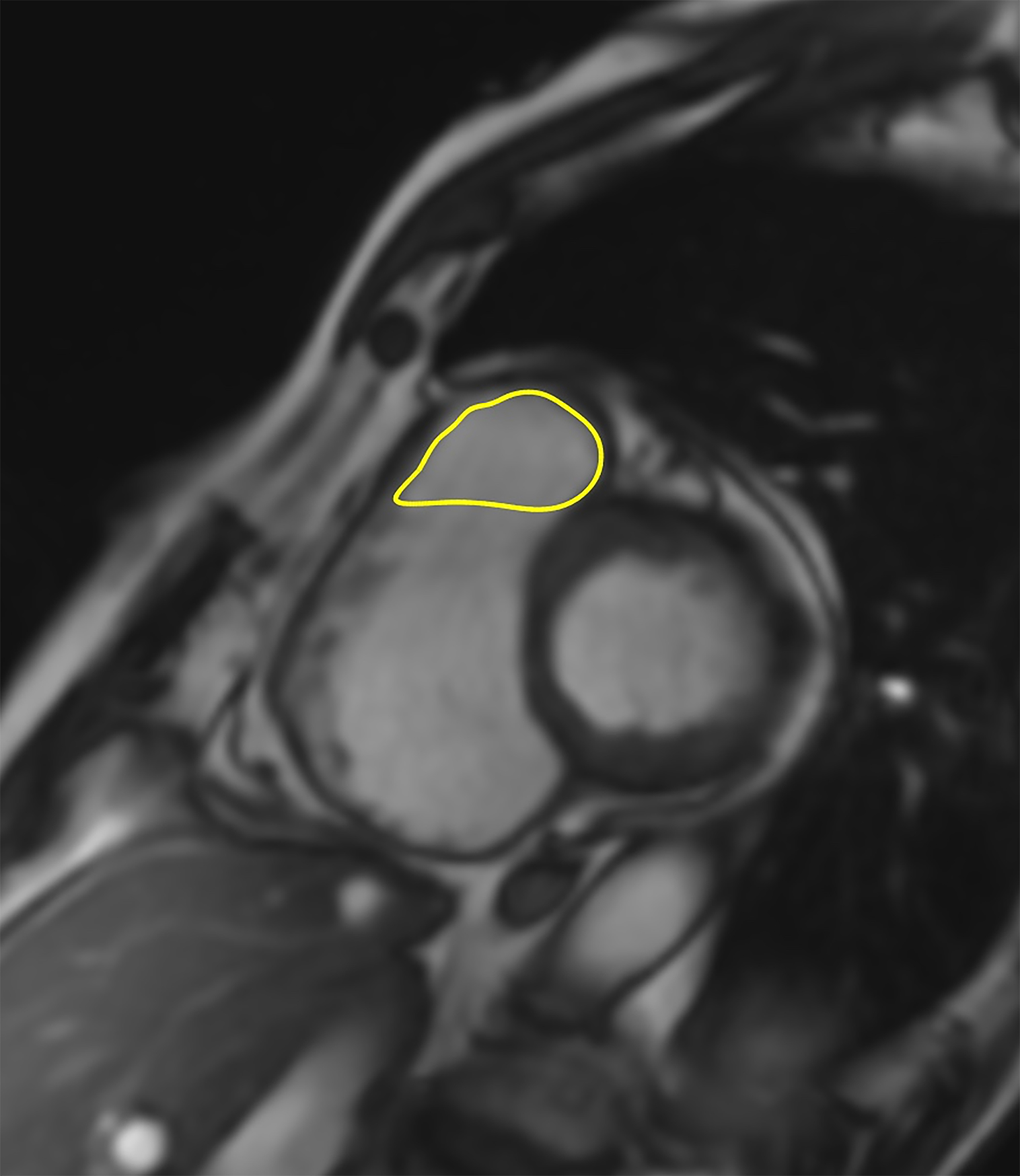

Supplement: S1 Fig — (TIFF) [file pone.0195594.s003.tiff]

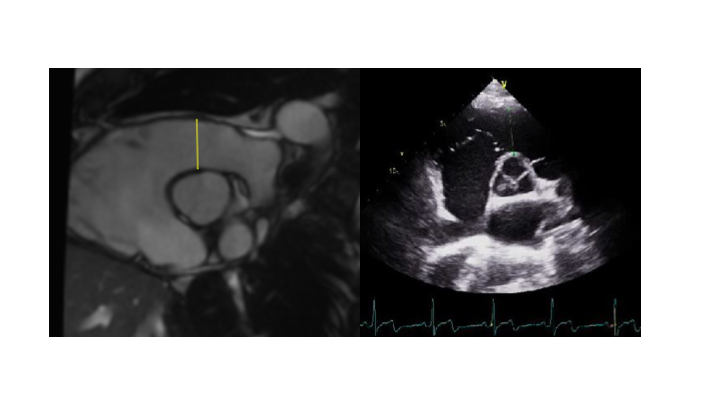

Supplement: S2 Fig — Example of right ventricular outflow tract measurement using (A) cardiac magnetic resonance imaging and (B) echocardiogram. (TIFF) [file pone.0195594.s004.tiff]
