# Supplementary material for: Relations between right ventricular morphology and clinical, electrical and genetic parameters in Brugada Syndrome
Source: PLoS One. 2018 Apr 13;13(4):e0195594. doi: 10.1371/journal.pone.0195594 (PMC5898761; doi:10.1371/journal.pone.0195594)
Supplement: S1 Text — (DOCX) [file pone.0195594.s005.docx]

**S1-SUPPLEMENTARY METHODS:**

Vertical long axis, four-chamber, RVOT (in sagittal and coronal planes) and short axis views of the heart were acquired over the entirety of both ventricles (9-12 slices) using retrospectively gated steady-state free precession cine MRI images. Image parameters: TR=3.2ms; TE=1.6 ms; flip angle=78**°**; slice thickness=10mm; matrix=192 x 256; field of view= 300-380 mm; and temporal resolution=40 ms, acquired during single breath-hold.

Manual segmentation of short-axis cine images with endocardial outline at end diastole and end systole were used to assess RV volumes (performed in Osirix software). Simpson’s rule was used to calculate the RV volume and RV ejection fraction. RVOT volume was calculated similarly in end-diastole by contouring the fibrous RVOT from the pulmonary valve leaflets superiorly, to the fibrous-muscular transition zone within the RVOT anteriorly based on delayed enhancement studies (described below). Posteriorly the fibrous RVOT was contoured to include volume until the superior aspect of the interventricular septum. An arbitrary line was contoured between the lower anterior and posterior landmarks to define the lower border zone of the fibrous RVOT (Supplementary Figure 1). Fibrous RVOT measurements were independently analysed in all subjects by two observers with an intra-class correlation coefficient of 0.923 for RVOT volumetric analysis. Simpson’s rule was used to calculate the RVOT volume.

The RVOT stack was performed with 4 to 5 contiguous slices. The slice which demonstrated the centre of the aortic valve en-face and the pulmonary valve in its long axis was chosen for measurement of RVOT dimension. This was measured at end-diastole in a line perpendicular to the plane of RVOT from the centre of the aortic valve to anterior wall of RVOT (Supplementary figure 2 below). RVOT contractile function was visually assessed in two orthogonal planes by two experienced observers and categorised as either normally contractile or dyskinetic or akinetic.

Segmented phase-sensitive inversion recovery sequences (Imaging parameters: TR= 2 x RR interval; TE= 3.4 ms; flip angle=25**°**; slice thickness=10mm; matrix=144 x 256, FOV = 300-380mm, acquired during a single breath hold) were used to identify myocardial scar 10 min post administration of intravenous contrast (0.1 mmol of Gadobutrol, Gadovist). Dedicated short-axis slices and RVOT views were performed to identify RV myocardial and RVOT scar.
