# Supplementary material for: Relations between right ventricular morphology and clinical, electrical and genetic parameters in Brugada Syndrome
Source: PLoS One. 2018 Apr 13;13(4):e0195594. doi: 10.1371/journal.pone.0195594 (PMC5898761; doi:10.1371/journal.pone.0195594)
Supplement: S2 Text — (PDF) [file pone.0195594.s006.pdf]

| MRI or echo | Study code | LVESV | Indexed LVESV | LVEDV | Indexed LVEDV | LV SV |
|---|---|---|---|---|---|---|
| 1 | 1 | 69 | 31 | 140 | 63 | 71 |
| 1 | 3 | 24 | 12 | 100 | 52 | 76 |
| 1 | 5 | 71 | 37 | 150 | 77 | 79 |
| 1 | 7 | 53 | 26 | 143 | 70 | 90 |
| 1 | 12 | 48 | 23 | 148 | 75 | 100 |
| 1 | 17 | 44 | 25 | 119 | 69 | 75 |
| 1 | 18 | 70 | 42 | 157 | 93 | 87 |
| 1 | 19 | 62 | . | 186 | . | 125 |
| 1 | 20 | 58 | 27 | 156 | 71 | 98 |
| 1 | 23 | 25 | 12 | 85 | 42 | 60 |
| 1 | 26 | 58 | 36 | 136 | 83 | 78 |
| 1 | 31 | 34 | 24 | 98 | 70 | 64 |
| 1 | 32 | 47 | 21 | 173 | 76 | 126 |
| 1 | 33 | 37 | 23 | 124 | 77 | 87 |
| 1 | 36 | 39 | 21 | 123 | 68 | 84 |
| 1 | 37 | 49 | 24 | 143 | 69 | 94 |
| 1 | 42 | 71 | 37 | 145 | 76 | 74 |
| 1 | 43 | 54 | 24 | 156 | 68 | 102 |
| 1 | 44 | 25 | 13 | 98 | 53 | 73 |
| 1 | 46 | 34 | 19 | 86 | 48 | 52 |
| 1 | 47 | 80 | 40 | 164 | 82 | 84 |
| 1 | 49 | 22 | 13 | 62 | 38 | 40 |
| 1 | 50 | 31 | 16 | 84 | 44 | 53 |
| 1 | 51 | 33 | 19 | 87 | 49 | 54 |
| 1 | 52 | 48 | 27 | 146 | 82 | 98 |
| 1 | 53 | 44 | 25 | 109 | 62 | 65 |
| 1 | 54 | 35 | 18 | 148 | 76 | 113 |
| 1 | 55 | 57 | 26 | 187 | 86 | 130 |
| 1 | 61 | 57 | 27 | 137 | 65 | 80 |
| 1 | 69 | . | 42 | . | 54 | 62 |
| 2 | 4 | . | . | . | . | . |
| 2 | 8 | . | . | . | . | . |
| 2 | 10 | . | . | . | . | . |
| 2 | 21 | . | . | . | . | . |
| 2 | 22 | . | . | . | . | . |
| 2 | 25 | . | . | . | . | . |
| 2 | 30 | . | . | . | . | . |
| 2 | 40 | . | . | . | . | . |
| 2 | 41 | . | . | . | . | . |
| 2 | 71 | . | . | . | . | . |
| 2 | 72 | . | 41 | . | 146 | 105 |
| 2 | 66 | . | . | . | . | . |

| LVEF | LV CO | LV CI | RVESV | Indexed RVESV | RVEDV |
|---|---|---|---|---|---|
| 51 | . | . | 69 | 31 | 132 |
| 76 | 5.30 | 2.80 | 82 | 43 | 156 |
| 53 | 5.00 | 2.60 | 77 | 40 | 158 |
| 63 | 4.10 | 2.00 | 74 | 36 | 168 |
| 68 | . | . | 73 | . | 165 |
| 63 | 4.50 | 2.60 | 76 | 44 | 152 |
| 55 | 5.00 | 3.00 | 88 | 52 | 176 |
| . | 6.4 | 3.2 | . | . | . |
| 63 | 6.90 | 3.10 | 76 | 35 | 179 |
| 71 | 4.30 | 2.10 | 48 | 24 | 110 |
| 57 | 5.5 | 3.3 | 59 | 36 | 135 |
| 65 | . | . | 43 | 31 | 102 |
| 73 | 8.80 | 3.80 | 83 | 36 | 209 |
| 70 | 5.70 | 3.50 | 51 | 32 | 139 |
| 68 | 5.10 | 2.80 | 69 | 38 | 154 |
| 66 | . | . | 81 | 39 | 175 |
| 51 | 4.7 | 2.5 | 85 | 44 | 159 |
| 65 | 6.4 | 2.8 | 65 | 29 | 166 |
| 74 | 5 | 2.7 | 51 | 27 | 124 |
| 60 | 4.1 | 2.3 | 46 | 26 | 95 |
| 51 | 5.5 | 2.7 | 65 | 32 | 142 |
| 65 | . | . | 21 | 13 | 53 |
| 63 | . | . | 45 | 24 | 92 |
| 62 | 4.5 | 2.6 | 43 | 24 | 101 |
| 67 | . | . | 97 | 38 | 165 |
| 60 | . | . | 30 | 17 | 97 |
| 76 | 7.9 | 4.1 | 53 | 27 | 163 |
| 70 | 9.1 | 4.2 | 68 | 31 | 191 |
| 58 | . | . | 82 | 39 | 163 |
| 60 | . | . | . | 54 | . |
| . | . | . | . | . | . |
| . | . | . | . | . | . |
| . | . | . | . | . | . |
| . | . | . | . | . | . |
| . | . | . | . | . | . |
| . | . | . | . | . | . |
| . | . | . | . | . | . |
| . | . | . | . | . | . |
| . | . | . | . | . | . |
| . | . | . | . | . | . |
| 72 | . | . | . | 75 | . |
| . | . | . | . | . | . |

| Indexed RVEDV | RV EF | RV CO | RV CI | RVOT dimension GK | RVOT volume GK |
|---|---|---|---|---|---|
| 60 | 48 | . | . | 28 | 15 |
| 81 | 47 | 5.2 | 2.7 | 25 | 13 |
| 81 | 51 | 5.1 | 2.6 | 26 | 15 |
| 82 | 56 | 4.3 | 2.1 | 30 | 16 |
| . | 56 | . | . | 21 | 11 |
| 88 | 50 | 4.6 | 2.6 | 18 | . |
| 105 | 50 | 5.1 | 3.00 | 28 | 15 |
| . | . | . | . | 30 | 16 |
| 82 | 58 | 7.2 | 3.3 | 23 | 16 |
| 54 | 56 | 4.5 | 2.2 | 20 | 13 |
| 83 | 56 | 5.3 | 3.3 | 25 | . |
| 73 | 58 | . | . | 20 | 12 |
| 91 | 60 | 8.8 | 3.8 | 27 | 15 |
| 86 | 63 | 5.7 | 3.5 | 25 | 13 |
| 85 | 55 | 5.2 | 2.9 | 28 | 13 |
| 84 | 54 | . | . | 28 | 16 |
| 83 | 47 | 4.7 | 2.5 | 23 | 15 |
| 73 | 61 | 6.4 | 2.8 | 34 | 15 |
| 67 | 59 | 5 | 2.7 | 25 | 14 |
| 53 | 52 | 3.9 | 2.2 | 18 | 14 |
| 71 | 54 | 5 | 2.5 | 29 | 15 |
| 32 | 60 | . | . | 23 | 13 |
| 48 | 51 | . | . | 21 | 15 |
| 57 | 57 | 4.9 | 2.7 | 26 | 13 |
| 93 | 59 | . | . | 27 | 13 |
| 57 | 70 | . | . | 30 | 12 |
| 84 | 66 | 7.7 | 3.9 | 32 | 16 |
| 88 | 64 | 8.6 | 4 | 27 | 16 |
| 78 | 50 | . | . | 27 | 17 |
| 116 | 53 | . | . | 23 | 11 |
| . | . | . | . | . | . |
| . | . | . | . | 22 | . |
| . | . | . | . | . | . |
| . | . | . | . | 29 | . |
| . | . | . | . | 27 | . |
| . | . | . | . | 31 | . |
| . | . | . | . | 25 | . |
| . | . | . | . | . | . |
| . | . | . | . | 21 | . |
| . | . | . | . | 25 | 13 |
| 184 | 59 | . | . | 22 | 13 |
| . | . | . | . | . | . |

| RVOT motion | RV LGE | abnormal rvot |
|---|---|---|
| 1 | . | 2 |
| 2 | 0 | 2 |
| 2 | 0 | 2 |
| 1 | 0 | 2 |
| 1 | 0 | 1 |
| 1 | 0 | 1 |
| 1 | 0 | 2 |
| 2 | . | 2 |
| 1 | 0 | 1 |
| 2 | 0 | 2 |
| 2 | 0 | 2 |
| 1 | 0 | 1 |
| 1 | 0 | 2 |
| 1 | 0 | 1 |
| 1 | 0 | 2 |
| 1 | 0 | 2 |
| 2 | . | 2 |
| 1 | . | 2 |
| 1 | . | 1 |
| 1 | . | 1 |
| 1 | . | 2 |
| 2 | . | 2 |
| 2 | . | 2 |
| 1 | . | 2 |
| 1 | . | 2 |
| 1 | . | 2 |
| 1 | . | 2 |
| 2 | . | 2 |
| 1 | no | 2 |
| 1 | . | 1 |
| 1 | . | 1 |
| 2 | . | 2 |
| 1 | . | 1 |
| 1 | . | 2 |
| 1 | . | 2 |
| 1 | . | 2 |
| 2 | . | 2 |
| 1 | . | 1 |
| 1 | . | 1 |
| 1 | . | 1 |
| 2 | . | 2 |
| 1 | . | 1 |
